# Competition and niche construction in a model of cancer metastasis

**DOI:** 10.1371/journal.pone.0198163

**Published:** 2018-05-29

**Authors:** Jimmy J. Qian, Erol Akçay

**Affiliations:** Department of Biology, University of Pennsylvania, Philadelphia, PA, United States of America; Beijing Cancer Hospital, CHINA

## Abstract

Niche construction theory states that not only does the environment act on populations to generate Darwinian selection, but organisms reciprocally modify the environment and the sources of natural selection. Cancer cells participate in niche construction as they alter their microenvironments and create pre-metastatic niches; in fact, metastasis is a product of niche construction. Here, we present a mathematical model of niche construction and metastasis. Our model contains producers, which pay a cost to contribute to niche construction that benefits all tumor cells, and cheaters, which reap the benefits without paying the cost. We derive expressions for the conditions necessary for metastasis, showing that the establishment of a mutant lineage that promotes metastasis depends on niche construction specificity and strength of interclonal competition. We identify a tension between the arrival and invasion of metastasis-promoting mutants, where tumors composed only of cheaters remain small but are susceptible to invasion whereas larger tumors containing producers may be unable to facilitate metastasis depending on the level of niche construction specificity. Our results indicate that even if metastatic subclones arise through mutation, metastasis may be hindered by interclonal competition, providing a potential explanation for recent surprising findings that most metastases are derived from early mutants in primary tumors.

## Introduction

A cancer tumor is a collection of abnormal cells whose unregulated proliferation damages surrounding host tissue, often resulting in patient death. It is also a population of genetically and phenotypically diverse cells that compete, propagate, and contribute (or not) to the cellular society. Tools from population biology are therefore increasingly used to study cancer dynamics. Cancer’s genetic instability and high mutation rate, compounded with harsh spatial constraints, a dearth of nutrients, and immune surveillance, lead to rapid selection for the survival of the fittest tumor cells. However, the evolutionary dynamics of tumors are only fully comprehensible when the ecological context—the tumor ecosystem—is considered [1, 2]. This entails applying ecological concepts such as predation, niches, and invasion (in evolutionary theory and in this paper, “invasion” refers to the establishment of a mutant genotype into an existing population, a concept distinct from cancer “invasion,” or expansion, into surrounding tissue). Accordingly, a number of ecological models have provided useful insight into cancer progression [3, 4].

A recently influential idea in ecology is that not only does the environment act on a population to generate selection pressures and Darwinian evolution, but organisms reciprocally modify the environment through a process called niche construction (also known as ecological engineering) [5, 6]. Via niche construction, organisms not only influence aspects of the ecosystem such as resource flow and trophic relationships, but they modify the actual sources of natural selection acting on themselves and their neighbors. For example, new selection pressures on beavers’ teeth, tail, and social behavior arise due to the construction of a dam [6]. The environmental modifications resulting from niche construction may be passed down to descendants through ecological inheritance, which has been recognized as a key aspect of extra-genetic inheritance [7].

Niche construction also likely plays an important role in cancer population biology [8–11]. Cancer cells greatly alter their microenvironments. For example, tumor cells release angiogenic factors such as vascular endothelial growth factor and stimulate vascularization [12–14], reduce local pH [15], release a gamut of growth factors such as insulin-like growth factor II [16], and secrete matrix metalloproteinases that degrade extracellular matrix proteins [12]. Tumors also drastically alter the local flow of nutrients and signaling factors, creating a nutrient-poor ecosystem that is passed down to descendant cells via ecological inheritance. This ecological inheritance promotes tumor cell heterogeneity and cancer growth, suggesting that cancer niche construction may be a worthwhile therapeutic target [8].

In this paper, we use niche construction theory to examine metastasis. Metastasis is not simply a result of mutation of tumor subclones into more invasive phenotypes and subsequent cell dissemination; it additionally requires the construction of a pre-metastatic niche [10, 17–22]. The concept of the pre-metastatic niche dates back to Paget’s “seed and soil” hypothesis, which states that tumors (the “seed”) are predisposed to metastasize to certain organs (the “soil”) because the metastatic site must provide a milieu conducive to the recruitment and settlement of disseminated tumor cells [23]. This receptive microenvironment, termed the pre-metastatic niche, must be established before metastasis can occur [10, 17–22]. Examples of pre-metastatic niche construction include increasing vascular permeability and clot formation, altering local resident cells such as fibroblasts, remodeling the extracellular matrix, and activating and recruiting non-resident cells such as haematopoietic progenitor cells and other bone marrow-derived cells, which further induce many subsequent changes [17]. Interestingly, evidence has shown that primary tumors actively prepare distant organs for reception of future metastatic cells by secreting various factors and extracellular vesicles that foster pre-metastatic niche construction into the bloodstream [17–22, 24–29]. Primary tumor-derived secretions that promote pre-metastatic niche construction include TGF*β* [18, 21], TNF-*α* [18], placental growth factor [18, 22], vascular endothelial growth factor [18, 21, 22], lysyl oxidase [19], microvesicles [29], exosomes [20, 26–28], and many more [17, 18]. These findings show that some primary tumor cells sacrifice metabolic resources in order to promote successful settlement by their disseminated descendants into metastatic sites, which provides no benefit to themselves. Why such behavior is so common is an interesting question especially because the ability of a tumor to metastasize cannot evolve adaptively analogous to life-history traits, since tumors are not selected to metastasize between generations and cancer lineages are in general evolutionary dead-ends [2]. Accordingly, the ability to metastasize, when it does occur, arises as a result of local ecological dynamics of a tumor. In this paper, we are interested in the fate of primary tumor mutations that promote pre-metastatic niche construction, rather than the entire metastatic cascade or settlement into the metastatic site.

Although previous work has recognized the applicability of niche construction theory to cancer [8, 9], there are only a few formal models of the phenomenon. Among these, Bergman and Gligorijevic [10] proposed a framework to integrate experimental metastasis data with niche construction theory, with the goal of providing a predictive model that can be directly parameterized. Another model by Gerlee and Anderson [30] studied the evolution of tumor carrying capacity as a function of niche construction. They assumed that niche construction increases the tumor carrying capacity, a phenomenon commonly seen in ecological settings. They noted that tumors may include both producers, which actively contribute to niche construction, and cheaters, which reap the benefits of niche construction without paying the growth rate cost of production. They showed that the specificity of the benefits from niche construction as well as spatial structure maintains selection for producers and allows for coexistence of cheaters and producers.

Another idea that motivates our model is the recent observation that metastatic cell lineages tend to diverge from the primary tumor early on [31]. In other words, metastasis involves mutations that occur early in the tumor’s lifetime. This finding contradicts the linear progression model of cancer, where metastatic tumors arise from late-stage primary tumors. This finding is somewhat paradoxical, since later-stage primary tumors are bigger and therefore harbor more mutations from which metastatic tumors might arise, and hence one might expect more metastatic tumors to be derived from late-stage tumors. As we will see below, competition between local and pre-metastatic niche constructors may provide a potential answer to this paradox.

We present a mathematical model of niche construction and metastasis in cancer. Our model contains producers of both the primary tumor (i.e., local) niche and the pre-metastatic niche, as well as cheaters. We model a tumor population with a carrying capacity that increases with local niche construction. We derive expressions for the ecological conditions necessary for metastasis, showing that they depend on niche construction specificity and the interclonal competition structure. Our results reveal a robust trade-off between the arrival of metastasis-promoting mutants and their ability to invade a tumor. Tumors composed only of cheaters remain small but are susceptible to invasion by cells that construct the pre-metastatic niche, whereas larger tumors containing producers may be unable to facilitate metastasis depending on the level of niche construction specificity. In certain competition structures, tumors containing only local producers can completely preclude metastasis unless invasion of metastasis-promoting subclones occurs early on. Our results highlight the fact that metastasis requires both the necessary genetic mutations and a suitable ecological milieu: even if metastatic subclones arise through mutation, invasion may not be possible due to competitive exclusion and a lack of niche opportunities. These findings can explain the observation that metastasis involves early mutations [31].

## Methods

We consider a primary tumor with *N* cells, which can include both producers and cheaters, and a bloodstream into which tumor cells can enter via intravasation. (An extended form of the model is discussed in S1 Appendix.) Producers participate in niche construction at a cost to their growth rate, since it takes energy and metabolic resources to secrete angiogenic factors, growth factors, and matrix metalloproteinases. Cheaters do not participate in niche construction but still benefit from it, so they have a higher growth rate than producers. We assume that a cell’s type (producer or cheater) is determined genetically.

There are three subsets of producers. Local producers contribute only to niche construction in the tumor’s immediate microenvironment, benefiting primary tumor cells but not circulating or metastasized cells. The extent of local niche construction is represented by the amount of resource *R*, a general resource that for example could represent the amount of recruited vasculature. The primary tumor also includes secondary producers, which contribute to the spatially distant pre-metastatic niche by secreting chemokines, growth factors, and exosomes into the bloodstream to allow circulating tumor cells to settle down to form a secondary tumor, as mentioned in the introduction. These molecules are carried away from the primary tumor and provide no benefit to primary tumor cells, so construction of the pre-metastatic niche is not included in the variable *R*. Secondary producers pay a growth cost similar to primary producers, but they otherwise act as cheaters from the primary tumor’s point of view since they benefit from *R* without contributing to it. Additionally, there are global producers that contribute to niche construction in both the primary microenvironment and the pre-metastatic niche and pay double the growth rate cost. Because pre-metastatic niche construction is required for metastasis, as discussed above, we treat the existence of secondary or global producers as a necessary condition for metastasis, consistent with our focus on interrogating the prerequisites of metastasis within the primary tumor. S2 Appendix discusses how our model is robust to changes in the interpretation of the four cell types and shows how our model may be generalized without changing the mathematical details or results.

**Fig 1:**
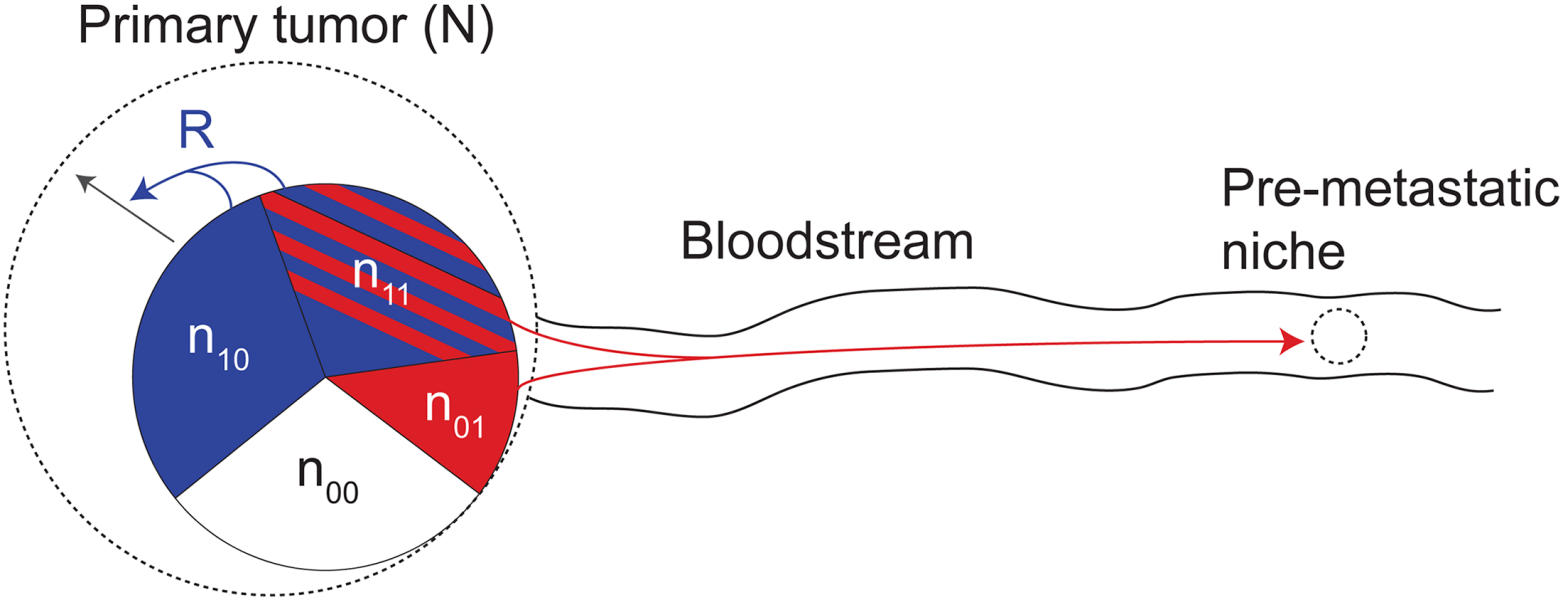
Schematic representation of the model. The model considers a primary tumor with four cell types and a distant pre-metastatic niche. Cheaters are white, local producers are blue, secondary producers are red, and global producers are both red and blue. Niche construction occurs in the primary microenvironment through production of resource *R*, which benefits the tumor by increasing carrying capacity, represented as a dotted line. Construction of the pre-metastatic niche by primary tumor cells is represented by the red arrow.

Cells are given a subscript (*x*, *y*), where *x* ∈ {0, 1} describes participation in local niche construction (0 for cheaters and 1 for producers) and *y* ∈ {0, 1} similarly denotes participation in pre-metastatic niche construction. The population of each cell type is *n*_*x*,*y*_ with respective growth rates *r*_*x*,*y*_. Local and global producers increase *R* with rate *g* and *R* suffers independent resource depletion with rate *l*. Fig 1 shows a schematic representation of the model.

Primary tumor cells enter the bloodstream as a result of intravasation. Local crowding has been suggested to cause a reduction in tumor cell fitness and lead to increased mutation rate and ecological dispersal [8]. Other studies have provided evidence that haematogenous tumor cell dissemination can begin early during primary tumor development and progression [21, 32, 33]. To account for these results and the ecological dispersal hypothesis, we introduce a function *m*(*N*, *R*) representing the rate at which primary tumor cells exit the local niche and enter the bloodstream. We assume this function has the form $m\left(N,R\right)=\frac{\alpha N}{k+\beta R(t)}$ where *α* is a constant and the denominator is the carrying capacity (discussed below). Cells tend to migrate more when they receive less of the share of resources in the microenvironment [8, 34–37]. It is important to note that the precise form of this dispersal function is not crucial to our results, because parameter estimation (see S3 Appendix) suggests *α* is several orders of magnitude smaller than any other parameter, a fact we use in simplifying our results as described later.

### Carrying capacity

We assume carrying capacity increases linearly with niche construction. Primary and secondary tumors possess intrinsic carrying capacity *k*, which can represent the number of cells that can survive without significant self-induced angiogenesis or release of growth factors. In the primary tumor, the carrying capacities of cheaters and secondary producers are both *k*+*β*_0_ *R*(*t*) while those of primary and global producers are *k*+*β*_1_ *R*(*t*). *β*_0_ and *β*_1_ are constants describing the benefit that either cheaters or producers receive from niche construction. If *β*_0_ ≠ *β*_1_, then either cheaters or producers use the resource more efficiently. This is analogous to the specificity of niche construction in Gerlee and Anderson’s model [30]. If $\frac{\beta_{1}}{\beta_{0}}>1$, modifications of the niche are specific to the genotype that generates it and cheaters are less able to free-ride. Strong specificity refers to $\frac{\beta_{1}}{\beta_{0}}\gg 1$.

### Competition

**Fig 2:**
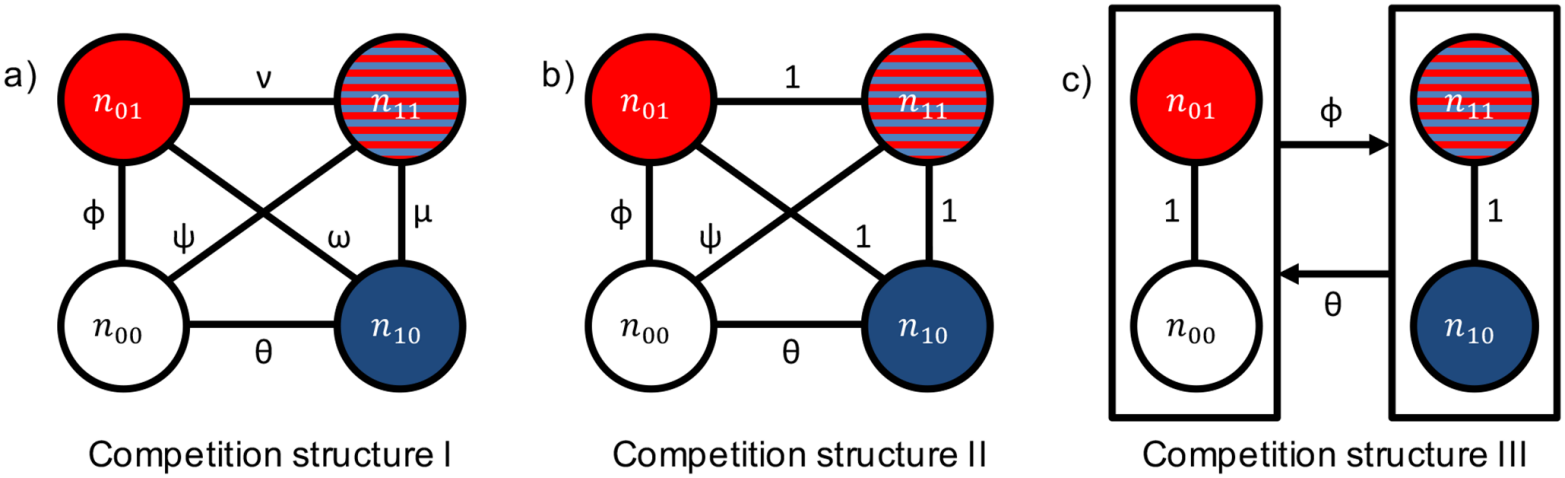
Schematic representation of the different competition structures. The strength of competition between each cell type is shown along connections in the lattice. Intraclonal competition is 1 for all cell types. *ϕ*, *θ*, and *ψ* are positive and less than 1. *ν*, *μ*, and *ω* are positive and less than or equal to 1. In competition structure III, the two distinct niches are represented by boxes. Cells that cheat in the primary tumor experience competition of magnitude *θ* due to, and compete with magnitude *ϕ* with, cells that produce the primary resource.

We assume cells grow according to Lotka-Volterra competition equations, shown in Table 1 with the parameters summarized in Table 2. We consider multiple competition structures with varying competition strength among the four cell types, summarized in Fig 2. In each, the strength of inter-type competition between the four cell types (symmetric in competition structures I and II) is denoted by Greek letters whose values are positive and less than or equal to 1. The magnitude of intraclonal competition is 1, such that interclonal competition strength is weaker than or equal to intraclonal competition. Biologically, stronger intra-type competition can stem from spatial considerations since cellular neighbors tend to be of the same cell type. During competition for resources on a local spatial scale, cells therefore compete more strongly with members of the same type. Stronger intra-type competition can also arise because different cell types utilize other resources (that we do not explicitly model) differentially. Cells of different clones may focus on different cellular pathways and require a different profile of metabolic resources. For example, cheaters focus on cell division and require a significant commitment to nucleotide biosynthesis and genome duplication. Producers, on the other hand, focus on protein production. The assumption that intraclonal competition is equal to or stronger than interclonal competition is discussed in further detail in the Discussion.

**Table 1:** Model equations.

Primary cheaters	$\frac{dn_{00}}{dt}=r_{00}n_{00}\left(1-\frac{n_{00}+\phi n_{01}+\theta n_{10}+\psi n_{11}}{k+\beta_{0}R}\right)-mn_{00}$
Secondary producers	$\frac{dn_{01}}{dt}=r_{01}n_{01}(1-\frac{\phi n_{00}+n_{01}+\omega n_{10}+\nu n_{11})}{k+\beta_{0}R}-mn_{01}$
Primary producers	$\frac{dn_{10}}{dt}=r_{10}n_{10}\left(1-\frac{\theta n_{00}+\omega n_{01}+n_{10}+\mu n_{11}}{k+\beta_{1}R}\right)-mn_{10}$
Global producers	$\frac{dn_{11}}{dt}=r_{11}n_{11}\left(1-\frac{\psi n_{00}+\nu n_{01}+\mu n_{10}+n_{11}}{k+\beta_{1}R}\right)-mn_{11}$
Resource	$\frac{dR}{dt}=g\left(n_{10}+n_{11}\right)-lR$

Governing equations of the model under competition structure I, and the corresponding variables whose rates of change they describe. Time dependence of *n* and *R* have been suppressed for notational simplicity. Dependence of *m* on *N* and *R* has also been suppressed. The equations for competition structures II and III are shown in S2 and S3 Tables.

**Table 2:** Summary of model parameters.

Parameter/variable	Description
*n*_*xy*_, *r*_*xy*_	number and growth rate of *xy*-type cells
*β*_0_	benefit from niche construction for cheaters and 2° producers
*β*_1_	benefit from niche construction for local and global producers
*k*	intrinsic carrying capacity
*α*	intravasation rate
*θ*, *ϕ*, *ω*, *ψ*, *μ*, *ν*	interclonal competition terms (see Fig 2)
*g*	resource production rate
*l*	independent resource depletion rate

A summary of the model parameters, some of which are estimated as described in S3 Appendix.

Competition structure I is the most general symmetric case in which no assumptions are made about the relative magnitudes of the various interclonal competition terms. By starting with this most general case, we show our results are robust to parameterization. Competition structure II and III are less general, as certain assumptions are made about the relative competition strengths. In competition structure II, interclonal competition between producers is as strong as intraclonal competition, while cheaters compete less with all three producer types. This scenario may arise if primary and secondary niche construction require similar metabolic resources so all producers occupy the same ecological niche, whereas cheaters focus on their own division instead of ecological engineering. In other words, cheaters express a unique phenotype with different target metabolic resources and cellular pathways than the producer types. Competition structure III assumes the two distinct niches in the tumor ecosystem are producing and cheating in the primary tumor regardless of propensity for secondary resource production. From the primary tumor’s standpoint, cheaters and secondary producers may occupy the same niche since neither cell type participates in local niche construction, while local and global producers both do and thus occupy a distinct niche. Intra-niche competition is as strong as intraclonal competition, while inter-niche competition is weaker.

### Separation of timescales

Simulations of the model (shown in S2 Fig) show that, for reasonable parameters (inferred from the literature in S3 Appendix), cell populations equilibrate more quickly than the resource dynamics. The latter keep growing without reaching an equilibrium at timescales relevant to tumor growth (i.e. the lifespan of a human). This is biologically intuitive since niche construction processes such as microenvironment vascularization are generally slower than cell division. This allows us to make a separation of timescales argument. In particular, we consider the cell dynamics to be fast and the resource dynamics to be slow. We first analyze the fast-changing variables while treating the slow-changing variable as constant. In other words, we find the equilibria of the cell dynamics while holding *R* constant (we refer to these equilibria of the fast dynamics, which are functions of *R*, as “quasi-equilibria”). Then, we analyze the dynamics of the slow variable *R* while assuming the fast variables are at a quasi-equilibrium.

## Results

For each competition structure, we examine a primary tumor that initially consists of only cheaters and local producers. The conditions for metastasis are equivalent to the invasion conditions of secondary or global producers into this tumor, since pre-metastatic niche construction is required for circulating tumor cells to settle into a secondary site. For invasion of secondary producers, $\frac{dn_{01}\left(t\right)}{dt}$ must be positive if a small but nonzero number of secondary producers cells are suddenly added to the population (e.g. through mutation). For invasion of global producers, $\frac{dn_{11}\left(t\right)}{dt}$ must be positive if a small but nonzero number of global producers are suddenly added to the population. There are three possible non-trivial quasi-equilibria of a local tumor: cheaters only, local producers only, and coexistence. We determine the stability of each quasi-equilibrium and evaluate the invasion conditions for secondary and global producers. These results for each competition structure are outlined in Table 3 and considered in detail below.

**Table 3:** Summary of results.

Competition structure	I	II	III
Producer-only stability	$\theta >\frac{\beta_{0}}{\beta_{1}}$	$\theta >\frac{\beta_{0}}{\beta_{1}}$	$\theta >\frac{\beta_{0}}{\beta_{1}}$
Invasion of 2° producers	$\omega <\frac{\beta_{0}}{\beta_{1}}$	*β*_0_ > *β*_1_	$\theta <\frac{\beta_{0}}{\beta_{1}}$
Invasion of global producers	*μ* < 1	false	false
Cheater-only stability	false	false	false
Invasion of 2° producers	true (*ϕ* < 1)	true (*ϕ* < 1)	*r*_01_ > *r*_10_
Invasion of global producers	true (*ψ* < 1)	true (*ψ* < 1)	true (*ϕ* < 1)
Coexistence stability	[messy]	[messy]	[messy]
Invasion of 2° producers	*β*_1_(*ϕθ* − *ω*) + *β*_0_(*ωθ* − *ϕ* − *θ*^2^ + 1) > 0	$\frac{\phi +\theta (\theta -1)-1}{\phi \theta -1}>\frac{\beta_{1}}{\beta_{0}}$	false
Invasion of global producers	*β*_0_(*μθ* − *ψ*) + *β*_1_(*ψθ* − *μ* − *θ*^2^ + 1) > 0	(*ψ* − *θ*)(*θβ*_1_ − *β*_0_) > 0	(*ϕ* − *θ*)(*β*_1_ *θ* − *β*_0_) > 0

A comparison of the invasion conditions at and stability conditions of each quasi-equilibrium for each competition structure. The conditions for stability of coexistence are omitted because they are mathematically intractable, though numerical analysis showed stability can be easily achieved for various parameter combinations. It is assumed that at the producer-only and coexistence quasi-equilibria, *R* >> *k* while at the cheater-only quasi-equilibrium, *k* >> *R*.

Tumors containing producers have a large amount of resource, i.e. *R* >>*k*, since the producer-only and coexistence quasi-equilibria result in rapid resource accumulation. On the other hand, tumors starting with cheaters only have low *R*, i.e. *k* >> *R* since there is no niche construction. Additionally, *α* ≈ 0 in any sum since *α* is several orders of magnitude smaller than any other parameter (see S3 Appendix). We use these facts in simplifying the derivation of stability and invasion conditions. The trajectories the tumor can undergo depend on niche construction specificity and inter-type competition structure. We consider each possibility in detail below.

### Competition structure I

The invasion conditions for secondary producer and global producers are, respectively,

$$\begin{array}{c} n_{00}\left(\phi +\frac{\alpha }{r_{01}}\right)+n_{10}\left(\omega +\frac{\alpha }{r_{01}}\right)<k+\beta_{0}R \end{array}$$ (1)

$$\begin{array}{c} n_{00}\left(\psi +\frac{\alpha }{r_{11}}\right)+n_{10}\left(\mu +\frac{\alpha }{r_{11}}\right)<k+\beta_{1}R. \end{array}$$ (2)

For large *R* and small *α*, the producer-only quasi-equilibrium is stable when

$$\begin{array}{c} \theta >\frac{\beta_{0}}{\beta_{1}}\;. \end{array}$$ (3)

This inequality means that the higher the specificity of niche construction (measured by $\frac{\beta_{1}}{\beta_{0}}$) the less likely cheaters are able to invade the population. Secondary producers can invade the local producer-only tumor if

$$\begin{array}{c} \omega <\frac{\beta_{0}}{\beta_{1}}\;. \end{array}$$ (4)

This condition similarly means that the higher the niche construction specificity, the less likely the invasion of secondary producers. If *ω* < *θ*, there is a window of specificity where the producer-only tumor is resistant to invasion by cheaters but susceptible to invasion by secondary producers. On the other hand, global producers can invade the producer-only tumor if

$$\begin{array}{c} \mu <1. \end{array}$$ (5)

Thus, the stability and resistance to invasion of a tumor containing only producers depends on the strength of interclonal competition and may depend additionally on niche construction specificity.

Secondary producers can invade the coexistence quasi-equilibrium if

$$\begin{array}{c} \beta_{1}\left(\phi \theta -\omega \right)+\beta_{0}\left(\omega \theta -\phi -\theta^{2}+1\right)>0\;. \end{array}$$ (6)

For high niche specificity, this is satisfied when *ϕθ* > *ω*, which is unlikely given that competition between cheaters and any producer type is less than competition among producers. Global producers can invade a tumor at coexistence if

$$\begin{array}{c} \beta_{0}\left(\mu \theta -\psi \right)+\beta_{1}\left(\psi \theta -\mu -\theta^{2}+1\right)>0\;. \end{array}$$ (7)

Importantly, whether a metastasis-promoting subclone can invade a tumor containing both cheaters and local producers depends on both niche construction specificity and competition strength. Finally, it is easy to see from Eqs (1) and (2) that a cheater-only tumor (with *R* = 0 and *α* ≪ 1) can always be invaded by any producer cell type regardless of specificity, as long as *ϕ* and *ψ* < 1.

These results point to an interesting trade-off: cheater-only tumors offer no competitive obstacle to metastasis. However, they remain small due to the lack of niche construction, which constrains the number of mutations they might experience that can lead to secondary or global producer clones. In contrast, if local producers invade first the tumor grows bigger, increasing the arrival rate of mutations, yet simultaneously the invasion conditions for a secondary or global producer become more stringent so that the pre-metastatic niche may be precluded by competition. As we discuss below, this tension is even more apparent in other competition structures.

### Competition structure II

The invasion conditions for secondary and global producers are, respectively,

$$\begin{array}{c} n_{00}\left(\phi +\frac{\alpha }{r_{01}}\right)+n_{10}\left(1+\frac{\alpha }{r_{01}}\right)<k+\beta_{0}R \end{array}$$ (8)

$$\begin{array}{c} n_{00}\left(\psi +\frac{\alpha }{r_{11}}\right)+n_{10}\left(1+\frac{\alpha }{r_{11}}\right)<k+\beta_{1}R\;. \end{array}$$ (9)

Producer-only tumors can be invaded by secondary producers when

$$\begin{array}{c} \beta_{0}>\beta_{1}\;, \end{array}$$ (10)

i.e. when there is no niche specificity and cells that do not produce the resource must benefit from it more than cells that do. Global producers cannot invade the producer-only tumor under this competition structure.

At the coexistence quasi-equilibrium, invasion of secondary producers can occur if

$$\begin{array}{c} \frac{\phi +\theta (\theta -1)-1}{\phi \theta -1}>\frac{\beta_{1}}{\beta_{0}}\;. \end{array}$$ (11)

This condition is less likely to be true with increasing specificity. Global producers can invade when

$$\begin{array}{c} \left(\psi -\theta \right)\left(\theta \beta_{1}-\beta_{0}\right)>0\;. \end{array}$$ (12)

Both invasion conditions for tumors with coexistence depend on the strength of competition and niche specificity. The cheater-only tumor, on the other hand, is always vulnerable to invasion by any producer cell types, just like for competition structure I. The trade-off between mutant arrival and invasion is reproduced in this competition structure and is even more apparent since global producers cannot invade producer-only tumors. Once again, stability and invasion of tumors containing producers depend on competition strength and specificity while cheaters are generally susceptible regardless of specificity.

### Competition structure III

The invasion conditions for secondary producers and global producers are, respectively,

$$\begin{array}{c} n_{00}\left(1+\frac{\alpha }{r_{01}}\right)+n_{10}\left(\theta +\frac{\alpha }{r_{01}}\right)<\beta_{0}R+k \end{array}$$ (13)

$$\begin{array}{c} n_{00}\left(\phi +\frac{\alpha }{r_{11}}\right)+n_{10}\left(1+\frac{\alpha }{r_{11}}\right)<\beta_{1}R+k\;. \end{array}$$ (14)

The condition for stability of the local producer-only quasi-equilibrium is Eq (3), just like the other two competition structures. Global producers cannot invade producer-only tumors, while invasion of secondary producers is possible when

$$\begin{array}{c} \theta <\frac{\beta_{0}}{\beta_{1}}\;. \end{array}$$ (15)

As niche construction specificity increases, this condition is less likely to be true. This invasion condition is mutually exclusive with the stability of the quasi-equilibrium. If $\theta >\frac{\beta_{0}}{\beta_{1}}$ then the tumor remains at the stable producer-only quasi-equilibrium and is resistant to invasion by cheaters, global producers, and secondary producers. If $\theta <\frac{\beta_{0}}{\beta_{1}}$ the quasi-equilibrium is unstable and susceptible to invasion by cheaters or secondary producers. The larger the competition that secondary producers would experience from local producers, the more efficiently they must be able to use the resource in order to invade.

At the coexistence quasi-equilibrium, the invasion condition for secondary producers is

$$\begin{array}{c} r_{01}>r_{00}. \end{array}$$ (16)

This condition is never fulfilled since secondary producers pay a growth rate cost relative to cheaters. The condition for invasion of global producers is

$$\begin{array}{c} \left(\phi -\theta \right)\left(\beta_{1}\theta -\beta_{0}\right)>0. \end{array}$$ (17)

The coexistence quasi-equilibrium allows for invasion of cells that contribute to the pre-metastatic niche only if they also contribute to local niche construction and only under certain levels of interclonal competition and specificity. Even if the necessary mutations for genesis of secondary producers occur, ecological conditions prevent the invasion of the lineage. Coexistence of cheaters and local producers can obstruct successful metastasis through a failure of settlement into the pre-metastatic niche rather than a failure of intravasation.

On the other hand, the cheater-only quasi-equilibrium is unstable and always vulnerable to invasion by global producers. Secondary producers can invade if

$$\begin{array}{c} r_{10}<r_{01}, \end{array}$$ (18)

i.e. if primary producers grow more slowly than secondary producers, which may be satisfied since the growth rate cost of local niche construction can easily be higher than that of preparing the pre-metastatic niche, again highlighting the susceptibility of cheater-only tumors to invasion by all producers.

In short, for all competition structures we consider, tumors with cheaters only are easily invaded while tumors containing producers are more difficult to invade, with restrictions on competition strength and niche construction specificity. To confirm this tension between invasion and mutation, we simulated the tumor and resource dynamics starting with cheaters only. The mutation rate in cancer is estimated to be 2 × 10^−7^ per cell division per gene [38] and the cell cycle length is approximately one day for at least some cancers [39]. We thus use a daily mutation rate of 2 × 10^−7^ and assume 1 out of 1000 mutations creates (11) cells from (00) cells or (10) cells from (01) cells. We assume 1 out of 500 mutations creates (01) or (10) cells from (00) cells, or (11) or (00) cells from (10) cells, since these cellular transformations do not require as drastic a phenotypic alteration. These mutation probabilities are somewhat arbitrary but the trade-off is robust to the choice of specific mutation probabilities. We choose these specific probabilities only to illustrate this trade-off in a convenient manner.

**Fig 3:**
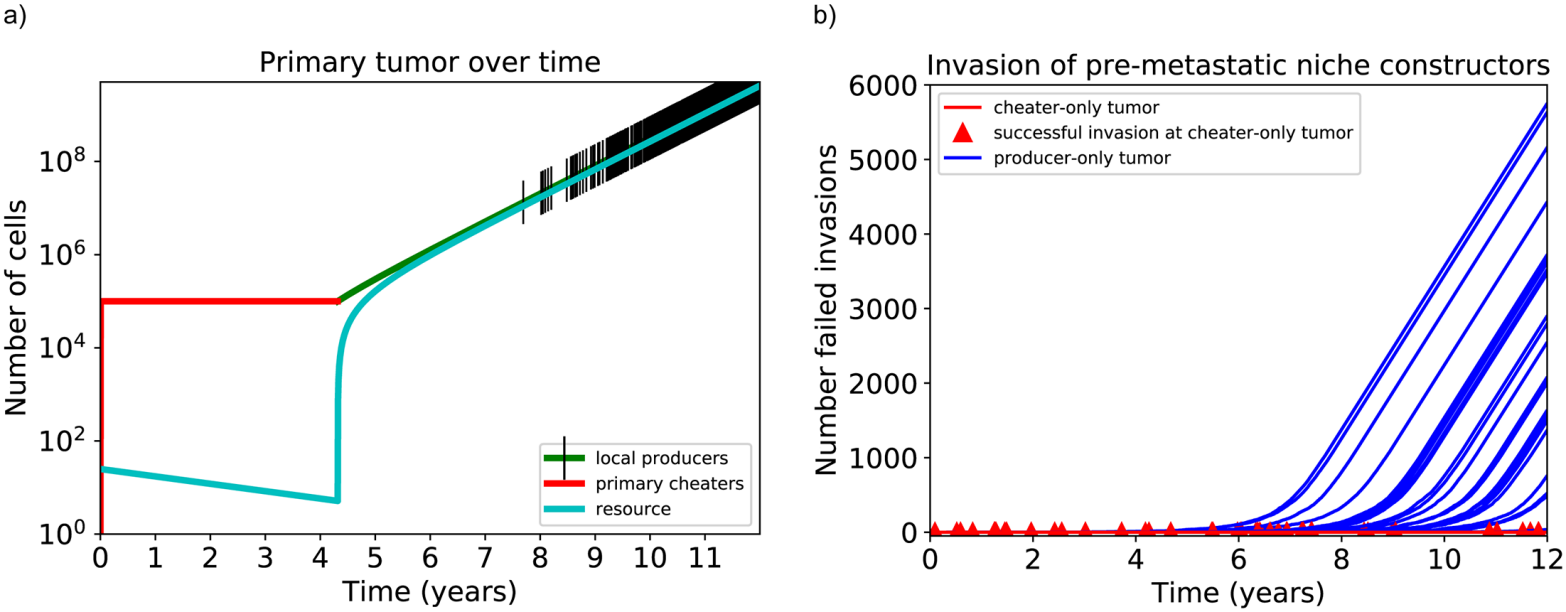
Simulation of tumors starting with cheaters. Parameters used are *r*_00_ = 0.07, *r*_10_ = 0.05, *r*_01_ = 0.045, *r*_11_ = 0.02, *k* = 10^5^, *β*_0_ = 1, *β*_1_ = 1.2, *θ* = *ϕ* = 0.9, *g* = 0.004, *l* = 0.001, *α* = 10^−6^, some of which are estimated in S3 Appendix. Mutation rates are mentioned in the text. If successful invasion of producers occurs, cheaters become extinct rather than arrive at coexistence for these parameters. A: Simulation of a single tumor starting with cheaters only and a small amount of resource. Black tick marks represent mutations leading to arrival of secondary or global producers, though none of them lead to successful invasion. B: Simulation of 200 tumors starting with cheaters only. Each red triangle indicates a successful invasion of a cheater-only tumor by secondary or global producers. Each blue curve represents a tumor that has been invaded by local producers; none of these producer-only tumors experienced successful invasion by secondary or global producers despite the arrival of numerous mutants, plotted on the y-axis.

Fig 3a shows a common tumor trajectory with clinically realistic tumor size. The tumor starts with cheaters and does not increase in size initially after arriving at the carrying capacity without producers. Once producers arise by mutation and successfully invade, cheaters go extinct. The tumor increases in size as resource production commences. The increasing size leads to numerous mutations, but these mutations do not lead to successful invasion since *R* has accumulated to a high level and we showed above that a stable producer-only quasi-equilibrium with high *R* is resistant to invasion. Fig 3b shows a clear trade-off between mutation rate and invasion. In tumors where producers arise from mutation and invade, size increases with time. The number of mutations increases drastically with tumor size, but these mutations all result in failed invasion. In tumors that remain cheater-only, successful invasion of secondary or global producers is possible, as shown by red triangles. There is a much smaller number of mutations for cheater-only tumors due to their small size, but once a mutation does arise, invasion is much more probable than in larger producer-only tumors.

### Tumor trajectories

**Fig 4:**
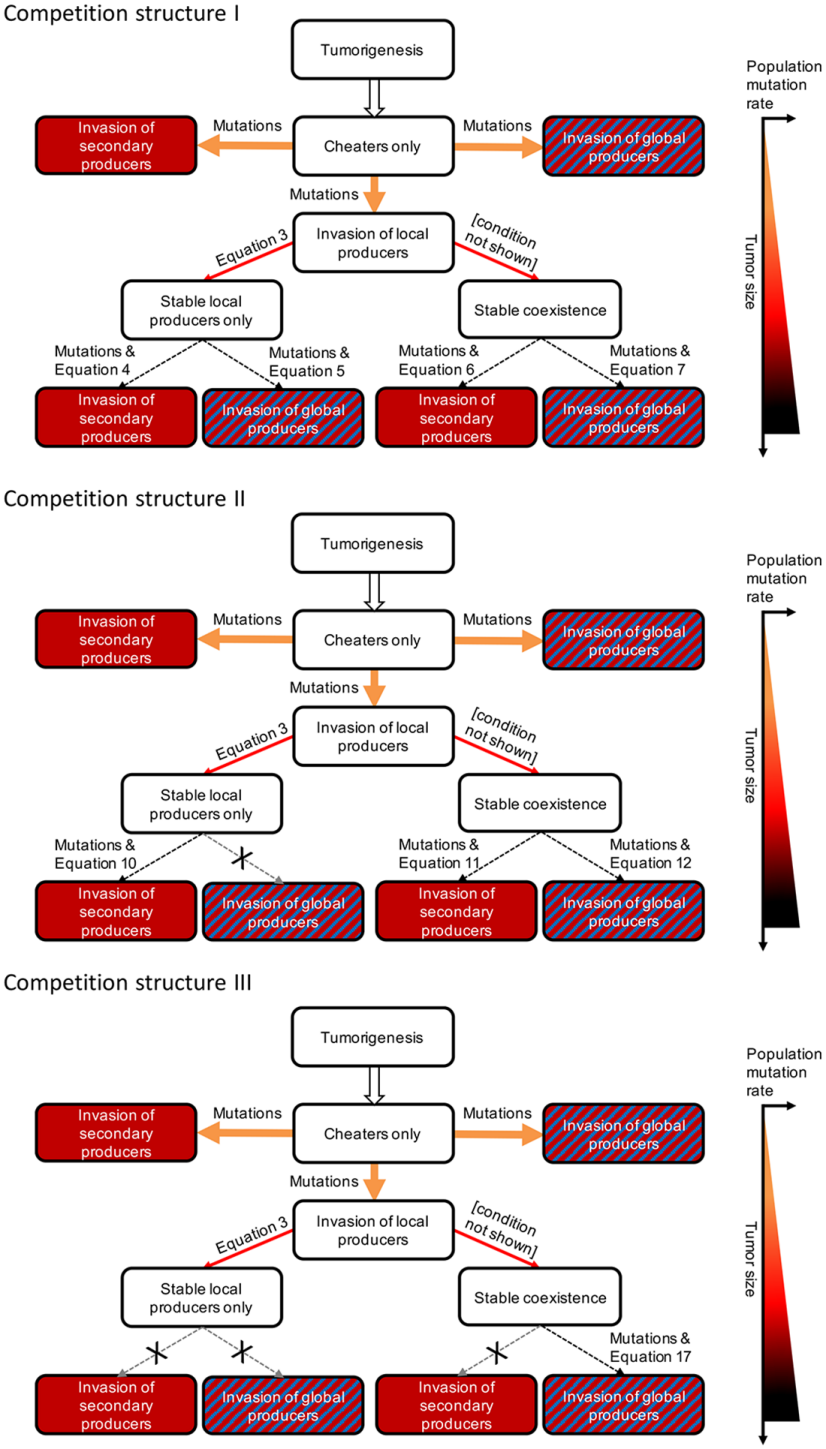
Schematic of possible tumor trajectories with their corresponding conditions. The thicker the arrow, the easier the ecological conditions are met. Arrow colors correspond to the mutation rate according to the mutation gradient on the right. Crossed out arrows indicate resistance to invasion. Tumor size and population mutation rate increase going down the flowchart, as indicated by the graph on the right.

Fig 4 schematically summarizes the trajectory tumors can undergo starting from cheaters only, in light of the results presented above. After tumorigenesis, cheaters proliferate and approach the intrinsic carrying capacity. The small initial tumor is always unstable and can be invaded by any producer cell type regardless of specificity. It can promote metastasis as long as the necessary mutations occur to generate secondary or global producers. However, to continue expanding the tumor population, niche construction is necessary. Mutations can lead to the appearance of producers from the cheater-only tumor, which saves the population from stagnation. Subsequent tumors reaching either coexistence or extinction of cheaters can, however, be resistant to invasion by metastasis-promoting lineages, depending on competition strength and niche construction specificity. Furthermore, under competition structure III, any tumor at the stable local producer-only quasi-equilibrium is resistant for all levels of specificity and any tumor containing coexistence is resistant to invasion by secondary producers.

## Discussion

We presented a simple model of niche construction in cancer, where local niche construction benefits all primary tumor cells by increasing the carrying capacity, and secondary niche construction (construction of the pre-metastatic niche) is needed for successful metastasis. Primary tumor cells can contribute to niche construction in one or both of the sites at a cost to their growth rate. Cheaters can reap the benefits of niche construction without paying the cost. Although no definitive information exists on the relative strengths of interclonal competition and density dependence, we have analyzed three plausible competition structures of varying generality.

The primary tumor, without any distant or global producers, can arrive at one of three nontrivial quasi-equilibria: extinction of cheaters, extinction of local producers, or coexistence of local producers and cheaters. The cheater-only quasi-equilibrium is vulnerable to invasion by any producer cell type, independent of niche construction specificity, as long as interclonal competition is weaker than intraclonal. On the other hand, quasi-equilibria containing producers have different requirements for stability and varying levels of susceptibility to the invasion of secondary or global producers, dependent on the strength of interclonal competition and niche construction specificity. The invasion of primary tumor cells that contribute to the pre-metastatic niche is a necessary condition for metastasis and settlement of the secondary tumor site [17–22]. Importantly, susceptibility or resistance to invasion are not intrinsic to a tumor, but are crafted through an ecological pathological relationship between the tumor and its microenvironment. Metastasis requires the necessary mutations for the genesis of certain subclones and also an ecological milieu that facilitates invasion of these subclones. Even if the appropriate mutations occur, the cells could fail to invade and instead die off if the tumor is resistant to invasion. We have shown that such resistance is more likely to occur in tumors containing producers, which are larger and accumulate more mutations. Small, cheater-only tumors experience fewer mutations yet are more able to facilitate the successful proliferation of metastasis-promoting lineages. Although we adopt a deterministic invasion perspective (i.e., mutant lineages either increase or not depending on the invasion condition), our argument also applies to the stochastic persistence of a small mutant lineage, since all things being equal, such persistence is less likely when invasion conditions are not satisfied.

Under all three competition structures, tumors containing only producers also demonstrate a trade-off between stability and the ability of secondary producers to invade. Regardless of interclonal competition strength, increasing niche construction specificity promotes stability of the producer-only tumor such that cheaters are unable to invade. This result agrees with Gerlee and Anderson’s findings that selection for niche construction requires sufficient specificity, as specificity keeps cheaters from free-riding [30]. However, we find that niche construction specificity makes it less likely that secondary producers can invade a producer-only tumor. This stems from the fact that secondary producers do not produce the primary resource and therefore are also selected against due to specificity of the resource. On the other hand, the ability of global producers to invade this tumor does not depend on niche construction specificity since they also produce the local resource and benefit with the same efficiency as local producers. Instead, invasion is possible only under competition structure I and only if interclonal competition between global and secondary producers is weaker than intraclonal competition.

Our main result is identifying a trade-off between the arrival of mutations leading to metastasis and their invasion success. This trade-off may help explain the early metastasis hypothesis, which posits that metastasis is not necessarily a late event in the tumor history, but rather can occur while the tumor is still small. Many genetic and clinical studies support this view [40]. For example, evidence suggests that cells in metastases are genetically less progressed in terms of tumor progression than primary tumor cells at diagnosis [41, 42] and that metastases do not necessarily not come from large tumors [43]. Studies of breast cancer metastasis suggest that it can be an early event [44–48]. Similarly, it has been proposed that metastatic capacity stems from mutations acquired early in a tumor history [49], an idea supported by a recent analysis of tumor phylogenies that shows early genetic divergence of metastatic lineages [31]. These observations contradict the idea that cancer follows a linear progression in which late-stage primary tumors facilitate metastasis. However, the idea that metastasis is not a late event may be paradoxical because late primary tumors are larger and harbor more mutations that can lead to the genesis of metastatic lineages. Our results indicate that this paradox and the early metastasis phenomenon may potentially operate through a tension between mutant arrival and invasion caused by competition between local and pre-metastatic niche constructors late in a tumor history. Secondary or global producers must invade while the tumor is still small, and if they do the pre-metastatic niche will begin recruiting circulating tumor cells from an early time point. Otherwise, the pre-metastatic niche may remain unprepared, since larger, late primary tumors containing producers may be resistant to invasion by pre-metastatic niche constructors. Large tumors participate in metastasis as long as invasion occurred while the tumor was still small. Accordingly, empirical evidence suggests that construction of the pre-metastatic niche is the limiting factor for establishing secondary tumors, not dissemination of circulating tumor cells which is independent of tumor size [43] and occurs starting early on [50]. This is supported by the parallel progression model of cancer, in which frequently disseminated cancer cells rarely establish themselves [31, 51]. In short, our results showing a tension between the arrival of a mutation for pre-metastatic niche construction and its successful establishment support the idea that metastasis begins early and provide a potential explanation for a paradoxical aspect of nonlinear tumor progression. Our conclusion that the timing of metastasis is partially mediated through the timing of invasion by pre-metastatic niche constructors into the primary tumor can be validated if empirical analyses reveal that mutations causing pre-metastatic niche construction occur before the divergence of metastatic tumor lineages from the primary tumor.

One implication of our results is that if certain types of cancers may be resistant to metastasis even over long periods of time despite the accumulation of mutations. This happens if the tumor switches to a producer-only or coexistence state with high niche-construction specificity and relatively high competition between different producer clones. Cancers that are not associated with metastasis are known since the work of Paget [23], who first proposed the “seed and soil” hypothesis. Our model suggests there could be an ecological, rather than genetic, explanation for the tendency of certain cancers to be less likely to metastasize. Resistance to invasion of metastatic subclones can be characteristic of particular cancers based on the typical cell types within the primary tumor cell population and the way they compete and use resources, rather than from a lack of necessary mutations.

Under competition structure III we find that in the coexistence quasi-equilibrium, reducing the growth rate of cheaters promotes the invasion of secondary producers. Chemotherapy is a method of targeting rapidly dividing cells and likely to disproportionately affect cheaters [52]. Thus, it is possible for chemotherapy to depress cheater growth rate enough such that *r*_00_ < *r*_01_, which would lead to Eq (16) to be satisfied and secondary producers to invade, a necessary step towards metastasis. This result is consistent with accumulating evidence that chemotherapy may increase the potential for metastasis by increasing pro-tumorigenic growth factors in the blood and mobilizing bone marrow-derived progenitor cells to make the secondary tumor site more receptive to circulating tumor cells [53–55].

The assumption that intra-type competition is stronger than inter-type competition is central to our results. This assumption, shared with other models of clonal dynamics [56, 57], can be viewed as an expression of the fact that cellular neighbors tend to be of the same cell type and competition for resources occurs on a local spatial scale. A wealth of mathematical and experimental evidence shows that tumors contain spatial clustering of subclones with relatedness decreasing as distance increases between cells [30, 58–64]. Another biological mechanism for stronger intraclonal competition is that different producer and cheater clones might occupy different niches, due to their different metabolic needs and utilization of different cellular pathways. Since cells that are highly related are more likely to use the same metabolic resources compared to cells that are less related, cells within the same clone compete with one another more strongly than they compete with a less-related cell of another clone that uses different resources and cellular pathways.

Another assumption we made was that the benefit of niche construction is manifested by increasing carrying capacity of both producers and cheaters [30]. Cancer cells thrive at cellular densities considerably higher than that of normal host cells [65]. Increased carrying capacity due to niche construction can be achieved through many mechanisms; perhaps the most obvious is angiogenesis. Tumors often live in highly acidic microenvironments due to their increased glycolytic metabolism. Inducing vascularization delivers oxygen, clears metabolic waste products, provides nutrients, and provides growth factors. It has been established that tumors larger than 1-2 mm are supported by newly formed blood vessels through secretion of various angiogenic factors, including PDGF (platelet-derived growth factor), AngI, AngII, and VEGF [66, 67]. One model used tumor carrying capacity as a function of blood vessel density due to the importance of tumor-induced angiogenesis [66], and this is essentially carrying capacity as a function of niche construction. Another example is the release of autocrine factors by tumor cells, since this increases their ability to divide despite high cell density [30]. In vitro [68] and in vivo [69] studies have observed tumors with a subset of producers that contributed to overall population growth through the secretion of diffusable growth factors. This is evidence that a tumor can have producers and cheaters with an increasing carrying capacity.

In summary, we have created a mathematical model to study metastasis as an outcome of niche construction. Though the full complexity of cancer dynamics and metastasis is not captured in this model, important guiding theoretical principles can be revealed in a simple “toy model” [70, 71]. The contribution of our toy model is to show a fundamental tension between mutant arrival and invasion. Tumors containing cheaters only are completely susceptible to invasion by all producer cell types while tumors containing producers can be resistant to invasion, dependent on competition strength and niche construction specificity. Our findings may help explain the early metastasis phenomenon and the observation that metastasis involves early mutations. We emphasize that successful metastasis requires a “double-hit” of the necessary genetic mutations and appropriate ecological conditions. Much research has focused on the genetic aspects of cancer initiation and progression, but this is insufficient if the context in which the genes exist and mutations arise is not considered [1, 4]. Paget’s “seed and soil” hypothesis is often invoked while studying metastasis [23]; our model shows that the analogy is more than evocative. Just as we need to consider the soil, sunlight, wind, and nearby flora and fauna to understand the germination of a seed, we also need to take the ecologist’s view to understand metastasis. Only then can we hope to stop the seed from spreading in the first place.

## Supporting information

S1 Appendix Extended model. (PDF)

S2 Appendix Alternative interpretation of cell types. (PDF)

S3 Appendix Estimation of parameters. (PDF)

S1 Fig Extended model schematic. Schematic representation of the extended mathematical model described in S1 Appendix. The model considers a primary tumor with four cell types, bloodstream with two cell types, and secondary tumor with two cell types. Cheaters are white and producers are blue. In the primary tumor, cells could additionally be secondary producers (red) or global producers (red and blue). Niche construction occurs in the tumor sites through production of resources *R*_1_ and *R*_3_, which benefit the tumors by increasing carrying capacity, represented as dotted lines. Construction of the pre-metastatic niche by primary tumor cells is represented by accumulation of resource *R*_2_, which facilitates settlement in the secondary tumor site.
(TIF)

S2 Fig Supplementary simulations. Simulation of a tumor with competition structure I starting with both cheaters and producers before the separation of timescales. *r*_00_ = 0.07, *r*_10_ = 0.05, *r*_01_ = 0.045, *r*_11_ = 0.02, *k* = 10^5^, *β*_0_ = 1, *β*_1_ = 1.2, *θ* = 0.9, *g* = 0.004, *l* = 0.001, *α* = 10^−6^. Simulations show that prior to the separation of timescales, the model (using competition structure I) contains a clinically realistic tumor size over time but fails to reach an equilibrium even after a decade. Different reasonable parameter combinations yield the same result. Cell populations in the model equilibrate more quickly than resource dynamics. The cell density always closely tracks the carrying capacity and the resource dynamics are slow. This allows us to make a separation of timescales argument, which is biologically expected since niche construction processes (such as microenvironment vascularization) are generally slower than cell division.
(TIFF)

S1 Table Extended model equations. Governing equations of the extended model described in S1 Appendix and the corresponding variables whose rates of change they describe, using competition structure I. Time dependence of *n* and *R* has been suppressed for notational simplicity. Dependence of *m* on *N*_1_ and *R*_1_ has also been suppressed.
(PDF)

S2 Table Governing equations of the model for competition structure II. (PDF)

S3 Table Governing equations of the model for competition structure III. (PDF)
