# Supplementary material for: Competition and niche construction in a model of cancer metastasis
Source: PLoS One. 2018 May 29;13(5):e0198163. doi: 10.1371/journal.pone.0198163 (PMC5973602; doi:10.1371/journal.pone.0198163)
Supplement: S1 Appendix — (PDF) [file pone.0198163.s001.pdf]

S1 Appendix: Extended model

The model we present in the main text can be extended to include dynamics in the bloodstream and secondary tumor site. We now consider three distinct ecosystems, denoted by subscripts 1, 2, and 3, respectively: the primary tumor site, the bloodstream, and a secondary site that receives metastatic cells to form a secondary tumor. Their cell populations are respectively N_1, N_2, and N_3 and can include both producers and cheaters. Cells are given a subscript (i, j), where $i \in \{0, 1\}$ describes the ability to participate in niche construction (0 for cheaters and 1 for producers) and $j \in \{1, 2, 3\}$ denotes which ecosystem the cell is in (1 for primary tumor, 2 for bloodstream, and 3 for secondary tumor). The number of each cell type is $n_{i,j}$ and the growth rate is $r_{i,j}$. A schematic of this extended model is shown in S1 Fig.

R_1 represents niche construction in the primary site. The extent to which the premetastatic niche has been constructed is measured by the amount of resource R_2. For the primary tumor, $i = hk$, where $h = 1$ indicates local production and $k = 1$ indicates distant production. Local and global producers increase R_1 with rate g_1 while secondary and global producers increase R_2 with rate g_2. R_1 and R_2 suffer independent resource depletion rates of l_1 and l_2, respectively.

Primary tumor cells enter the bloodstream as a result of intravasation and die with rate d due to immune surveillance, anoikis, or physical stress. The intravasation function has the same form as discussed in the main text, $m(N_1, R_1) = \frac{\alpha N_1}{k+\beta R_1(t)}$. Though there is no niche construction while migrating through the bloodstream, cells retain their propensity for local niche construction: local producers and global producers remain producers, while cheaters and secondary producers remain cheaters. We assume that once in the bloodstream, cells cease to engage in premetastatic niche construction, so we do not keep track of the distant producers and cheaters separately, reducing the number of cell types from four in the primary tumor to two in the bloodstream and secondary tumor: $(00, 1)$ and $(01, 1)$ cells become $(0, 2)$ cells in the bloodstream while $(10, 1)$ and $(11, 1)$ cells become $(1, 2)$ cells.

Cells can undergo extravasation and successfully settle the pre-metastatic niche as a function of R_2. We assume that construction of the pre-metastatic niche is necessary for circulating tumor cells to settle down. In particular, we postulate a linear relationship between settlement rate and R_2 with slope δ. Upon settling, $(0,2)$ cells become $(0,3)$ cells while $(1,2)$ cells become $(1,3)$ cells. There is local niche construction by $(1,3)$ cells in this metastatic tumor which increases resource R_3 with rate g_3. R_3 is a measure of local niche construction in the secondary tumor, which does not affect settlement but rather benefits cells that have already successfully metastasized. R_3 also has independent resource depletion with rate l_3. The equations of the extended model are summarized in S1 Table.
