# Supplementary material for: Competition and niche construction in a model of cancer metastasis
Source: PLoS One. 2018 May 29;13(5):e0198163. doi: 10.1371/journal.pone.0198163 (PMC5973602; doi:10.1371/journal.pone.0198163)
Supplement: S2 Appendix — (PDF) [file pone.0198163.s002.pdf]

S2 Appendix: Alternative interpretation of cell types

Our model includes four cell types: cheaters, local producers, secondary producers, and global producers. Local and global producers contribute to primary niche construction, while secondary and global producers contribute to pre-metastatic niche construction. This interpretation of the cell populations can actually be generalized: as long as cells pay some cost to promote metastasis, whether it be via pre-metastatic niche construction or some other mechanism, the mathematical details and results of our model remain the same. This is because we focus on the prerequisites of metastasis within the primary tumor. For the extended model in S1 Appendix, the settlement dynamics would change based on the interpretation of cell types.

We present one potential alternative interpretation of the four cell types. Local producers pay a cost to participate in local niche construction benefiting all primary tumor cells, but have low metastatic potential. Cheaters benefit from local niche construction without paying the cost, and also possess low metastatic potential. The third cell type, analogous to the original secondary producer, benefits from local niche construction without paying the cost, but has high metastatic potential which comes at a cost. The fourth cell type, analogous to the original global producer, participates in local niche construction at a cost and also possesses high metastatic potential which comes at a cost. This interpretation focuses not on the construction of the pre-metastatic niche, but rather on metastatic potential of primary tumor cells, without changing any of the model's mathematical details. In this framework, existence of cells with high metastatic potential is a prerequisite of metastasis. Metastatic potential can include various characteristics that promote the cell's ability to successfully spawn a metastatic lesion, for example the ability to evade numerous cell death signals that are induced by loss of attachment to neighboring cells (anoikis) and the extracellular matrix (amorphosis) [1]. It is reasonable to assume high metastatic potential may incur a growth rate cost in the primary tumor. For example, the motile invasive phenotype, which fosters metastasis, may be characterized by a growth rate cost [2], which may stem from the fact that cells capable of moving cannot divide while moving [3, 4]. In short, our mathematical

model is not sensitive to the specific interpretation of the cell types as long as there is a cost to promoting metastasis. In the main text, we focus on niche construction and the establishment of the pre-metastatic niche, but using other frameworks such as metastatic potential leads to the same results from the model.

References

[1] Mehlen P, Puisieux A. Metastasis: a question of life or death. Nature Reviews Cancer. 2006;6(6):449–458.

[2] Basanta D, Hatzikirou H, Deutsch A. Studying the emergence of invasiveness in tumours using game theory. The European Physical Journal B-Condensed Matter and Complex Systems. 2008;63(3):393–397.

[3] Giese A, Loo MA, Tran N, Haskett D, Coons SW, Berens ME. Dichotomy of astrocytoma migration and proliferation. International journal of cancer. 1996;67(2):275–282.

[4] Giese A, Bjerkvig R, Berens M, Westphal M. Cost of migration: invasion of malignant gliomas and implications for treatment. Journal of clinical oncology. 2003;21(8):1624–1636.
