# Supplementary material for: Competition and niche construction in a model of cancer metastasis
Source: PLoS One. 2018 May 29;13(5):e0198163. doi: 10.1371/journal.pone.0198163 (PMC5973602; doi:10.1371/journal.pone.0198163)
Supplement: S3 Appendix — (PDF) [file pone.0198163.s003.pdf]

S3 Appendix: Estimation of parameters

In order to keep the model as realistic as possible, we attempted to choose parameters such that the simulated tumor reflects some behaviors observed clinically. According to one study, breast tumors grow on average 7.8±3.9 years before detection, where the detection size is approximately 10^9 cells [1]. The value of k, the intrinsic carrying capacity, should reflect the fact that typical tumor population sizes can range from 10^6 to 10^{11} cells [2, 3]. One estimate is that the size limit of cancer cell populations prior to the initiation of angiogenesis is 10^5 [4], so this is a reasonable estimate for the intrinsic carrying capacity k. On the other hand, the size limit after angiogenesis is on the order of 10^{12} [4], which is generally viewed as the lethal tumor size at which patient death occurs [5]. Other methods estimate the maximum tumor size to be 12 cm [6] which corresponds to approximately 7.23×10^{11} cells [2], corroborating the 10^{12} estimate.

The intrinsic growth rates of clones can be estimated using data from the Norwegian Breast Cancer Screening Program [6]. The study used a logistic growth model and data from a large population to estimate doubling times of breast cancer tumors. In women aged 50-69 years, a 15 mm tumor doubled in diameter on average in 100 days while a 10 mm tumor doubled in diameter on average in 1.7 years. Using the conversion that one cubic centimeter tumor corresponds to 10^8 cells [2], and assuming tumors are perfect spheres, these two doubling times can be converted to 0.0707 and 0.0113 day^{-1}, taking into account size-dependent growth. Thus, a reasonable estimate for the highest growth rate r_{00} may be 0.07 day^{-1}. This agrees with growth rates obtained from other studies using clinical data [5] and is similar to assumed parameters used in another mathematical model [7].

The rate of intravasation into the bloodstream has been estimated to be on the order of 10^{-9} to 10^{-11} day^{-1}, but this seems to include the death rate of circulating cells [8]. It is often estimated that less than 1% of circulating tumor cells survive [9, 10]. Another estimate of the integrated rate of leaving the primary site and successfully joining a secondary tumor in pancreatic cancer is 6×10^{-7} per cell cycle [11]. We thus assume the parameter α describing intravasation is on the order of $\alpha = 10^{-6}$ day^{-1} or less, which is several orders of magnitudes

smaller than all other parameters.

References

[1] Speer JF, Petrosky VE, Retsky MW, Wardwell RH. A stochastic numerical model of breast cancer growth that simulates clinical data. Cancer Research. 1984;44(9):4124–4130.

[2] Del Monte U. Does the cell number 109 still really fit one gram of tumor tissue? Cell Cycle. 2009;8(3):505–506.

[3] Lipinski KA, Barber LJ, Davies MN, Ashenden M, Sottoriva A, Gerlinger M. Cancer evolution and the limits of predictability in precision cancer medicine. Trends in Cancer. 2016;2(1):49–63.

[4] Clare SE, Nakhlis F, Panetta JC. Molecular biology of breast metastasis The use of mathematical models to determine relapse and to predict response to chemotherapy in breast cancer. Breast Cancer Research. 2000;2(6):430.

[5] Spratt JA, Von Fournier D, Spratt JS, Weber EE. Decelerating growth and human breast cancer. Cancer. 1993;71(6):2013–2019.

[6] Weedon-Fekjær H, Lindqvist BH, Vatten LJ, Aalen OO, Tretli S. Breast cancer tumor growth estimated through mammography screening data. Breast Cancer Research. 2008;10(3):R41.

[7] Leder K, Holland EC, Michor F. The therapeutic implications of plasticity of the cancer stem cell phenotype. PloS One. 2010;5(12):e14366.

[8] Iskandar R. A Theoretical Model Of Breast Tumor Metastases In The Context Of Tumor Dormancy. 2016.

[9] Yang KR, Mooney SM, Zarif JC, Coffey DS, Taichman RS, Pienta KJ. Niche inheritance: a cooperative pathway to enhance cancer cell fitness through ecosystem engineering. Journal of Cellular Biochemistry. 2014;115(9):1478–1485.

[10] Fidler I. Selection of successive tumour lines for metastasis. Nature. 1973;242(118):148–149.

[11] Haeno H, Gonen M, Davis MB, Herman JM, Iacobuzio-Donahue CA, Michor F. Computational modeling of pancreatic cancer reveals kinetics of metastasis suggesting optimum treatment strategies. Cell. 2012;148(1):362–375.
