# Supplementary figures and images for: Competition and niche construction in a model of cancer metastasis
Source: PLoS One. 2018 May 29;13(5):e0198163. doi: 10.1371/journal.pone.0198163 (PMC5973602; doi:10.1371/journal.pone.0198163)

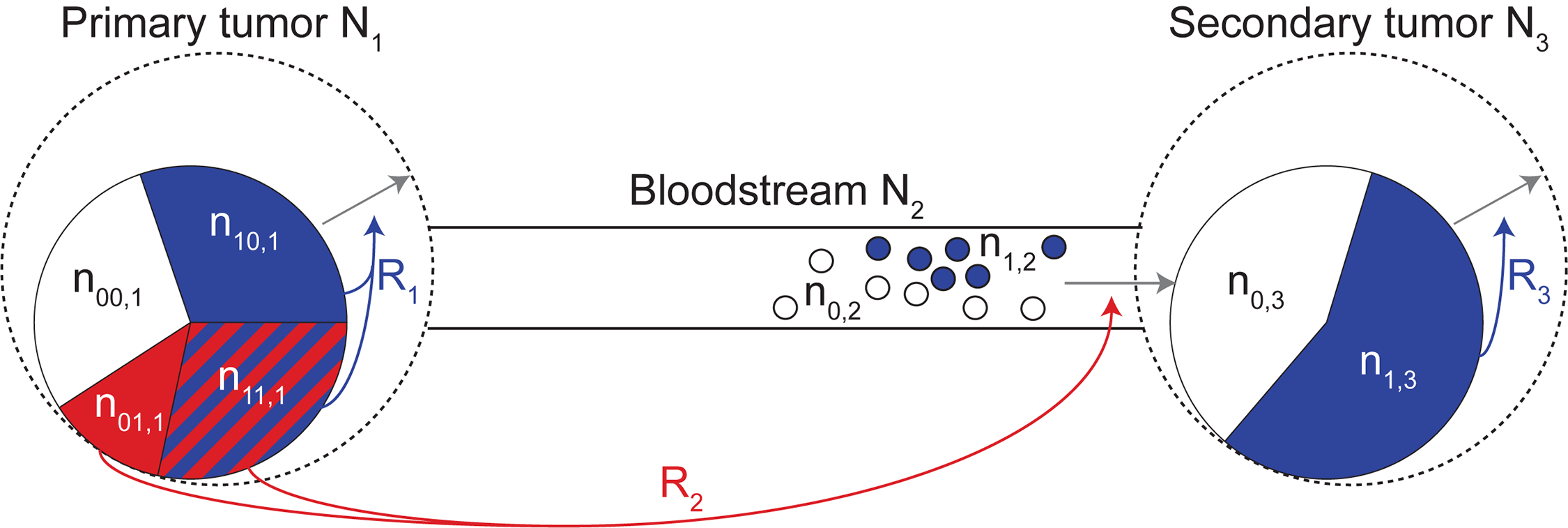

Supplement: S1 Fig — Schematic representation of the extended mathematical model described in S1 Appendix. The model considers a primary tumor with four cell types, bloodstream with two cell types, and secondary tumor with two cell types. Cheaters are white and producers are blue. In the primary tumor, cells could additionally be secondary producers (red) or global producers (red and blue). Niche construction occurs in the tumor sites through production of resources R1 and R3, which benefit the tumors by increasing carrying capacity, represented as dotted lines. Construction of the pre-metastatic niche by primary tumor cells is represented by accumulation of resource R2, which facilitates settlement in the secondary tumor site. (TIF) [file pone.0198163.s004.tif]

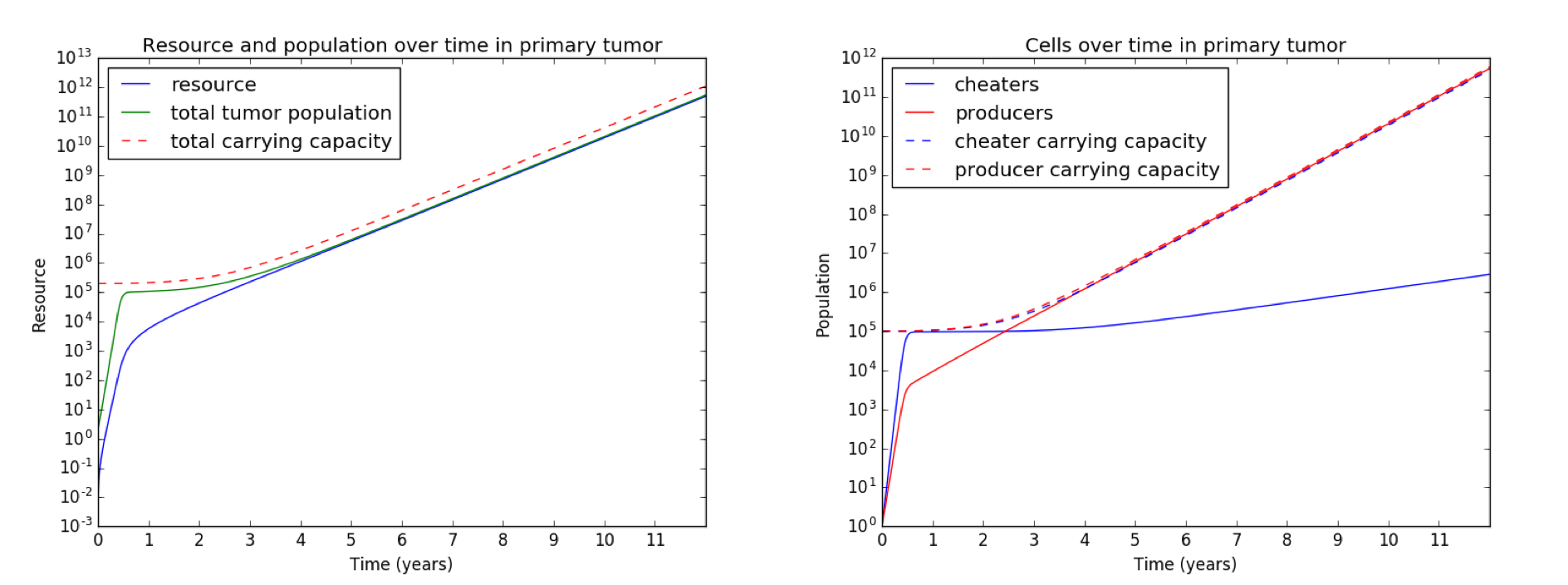

Supplement: S2 Fig — Simulation of a tumor with competition structure I starting with both cheaters and producers before the separation of timescales. r00 = 0.07, r10 = 0.05, r01 = 0.045, r11 = 0.02, k = 105, β0 = 1, β1 = 1.2, θ = 0.9, g = 0.004, l = 0.001, α = 10−6. Simulations show that prior to the separation of timescales, the model (using competition structure I) contains a clinically realistic tumor size over time but fails to reach an equilibrium even after a decade. Different reasonable parameter combinations yield the same result. Cell populations in the model equilibrate more quickly than resource dynamics. The cell density always closely tracks the carrying capacity and the resource dynamics are slow. This allows us to make a separation of timescales argument, which is biologically expected since niche construction processes (such as microenvironment vascularization) are generally slower than cell division. (TIFF) [file pone.0198163.s005.tiff]
