# Supplementary material for: Competition and niche construction in a model of cancer metastasis
Source: PLoS One. 2018 May 29;13(5):e0198163. doi: 10.1371/journal.pone.0198163 (PMC5973602; doi:10.1371/journal.pone.0198163)
Supplement: S1 Table — Governing equations of the extended model described in S1 Appendix and the corresponding variables whose rates of change they describe, using competition structure I. Time dependence of n and R has been suppressed for notational simplicity. Dependence of m on N1 and R1 has also been suppressed. (PDF) [file pone.0198163.s006.pdf]

S1 Table: Extended model equations

Primary cheaters	$\frac{dn_{00,1}}{dt} = r_{00,1}n_{00,1}\Big(1 - \frac{n_{00,1} + \phi n_{01,1} + \theta n_{10,1} + \omega n_{11,1}}{k + \beta_0 R_1}\Big) - mn_{00_1}$
Secondary producers	$\frac{dn_{01,1}}{dt} = r_{01,1}n_{01,1}\Big(1 - \frac{\phi n_{00,1} + n_{01,1} + \psi n_{10,1} + \nu n_{11,1}\big)}{k + \beta_0 R_1} - mn_{01,1}$
Primary producers	$\frac{dn_{10,1}}{dt} = r_{10,1}n_{10,1}\Big(1 - \frac{\theta n_{00,1} + \psi n_{01,1} + n_{10,1} + \mu n_{11,1}}{k + \beta_1 R_1}\Big) - mn_{10,1}$
Global producers	$\frac{dn_{11,1}}{dt} = r_{11,1}n_{11,1}\Big(1 - \frac{\omega n_{00,1} + \nu n_{01,1} + \mu n_{10,1} + n_{11,1}}{k + \beta_1 R_1}\Big) - mn_{11,1}$
Bloodstream cheaters	$\frac{dn_{0,2}}{dt} = \frac{\alpha N_1}{k + \beta_0 R_1}\big(n_{00,1} + n_{01,1}\big) - (d + \delta R_2)n_{0,2}$
Bloodstream producers	$\frac{dn_{1,2}}{dt} = \frac{\alpha N_1}{k + \beta_1 R_1}\big(n_{10,1} + n_{11,1}\big) - (d + \delta R_2)n_{1,2}$
Metastatic cheaters	$\frac{dn_{0,3}}{dt} = r_{0,3}n_{0,3}\Big(1 - \frac{n_{0,3} + \theta n_{1,3}}{k + \beta_0 R_3}\Big) + \delta R_2 n_{0,2}$
Metastatic producers	$\frac{dn_{1,3}}{dt} = r_{1,3}n_{1,3}\Big(1 - \frac{\theta n_{0,3} + n_{1,3}}{k + \beta_1 R_3}\Big) + \delta R_2 n_{1,2}$
Primary resource	$\frac{dR_1}{dt} = g_1\big(n_{10,1} + n_{11,1}\big) - l_1 R_1$
Settlement resource	$\frac{dR_2}{dt} = g_2\big(n_{01,1} + n_{11,1}\big) - l_2 R_2$
Metastasis resource	$\frac{dR_3}{dt} = g_3 n_{1,3} - l_3 R_3$
