# Supplementary material for: Competition and niche construction in a model of cancer metastasis
Source: PLoS One. 2018 May 29;13(5):e0198163. doi: 10.1371/journal.pone.0198163 (PMC5973602; doi:10.1371/journal.pone.0198163)
Supplement: S3 Table — (PDF) [file pone.0198163.s008.pdf]

S3 Table: Governing equations of the model for competition structure III

Primary cheaters	$\frac{dn_{00}}{dt} = r_{00}n_{00}\Big(1 - \frac{n_{00} + n_{01} + \theta\big(n_{10} + n_{11}\big)}{k + \beta_0 R}\Big) - mn_{00}$
Secondary producers	$\frac{dn_{01}}{dt} = r_{01}n_{01}\Big(1 - \frac{n_{00} + n_{01} + \theta\big(n_{10} + n_{11}\big)}{k + \beta_0 R}\Big) - mn_{01}$
Primary producers	$\frac{dn_{10}}{dt} = r_{10}n_{10}\Big(1 - \frac{\phi\big(n_{00} + n_{01}\big) + n_{10} + n_{11}}{k + \beta_1 R}\Big) - mn_{10}$
Global producers	$\frac{dn_{11}}{dt} = r_{11}n_{11}\Big(1 - \frac{\phi\big(n_{00} + n_{01}\big) + n_{10} + n_{11}}{k + \beta_1 R}\Big) - mn_{11}$
Resource	$\frac{dR}{dt} = g\big(n_{10} + n_{11}\big) - lR$
